# Editorials

**Published:** 1912-08

**Authors:** 


### EDITORIAL

The Business Office of the *Journal-Record* is 23 West Alabama Street.

The Editorial Office is 1014-15 Atlanta National Bank Building.

Address all Business Communications to *Journal-Record of Medicine,* 23 West Alabama Street.

Make remittances payable to *The Journal-Record of Medicine.*

On matters pertaining to the Editorial and Original Communications, address Edgar G. Ballenger, M. D., Atlanta, Ga.

Reprints of Original Articles will be furnished at cost price. Requests foı the same should always be made in THE MANUSCRIPT.

We will present, postpaid, on request to each contributor of an original article, twenty (20) marked copies of *The Journal-Record of Medicine* containing such article.

### THE GRACES IN MEDICAL LITERATURE.

While we seem to be right in the Golden Age of scientific research and achievement; while discoveries follow each other in quick succession, and while book after book comes to us laden with new and helpful knowledge, we cannot but notice, with few exceptions, one missing link in the harmonious whole, and that is the lack of seeming effort to clothe these valuable contributions in nice garments of English.

If one would read from that masterpiece of literature, Meig's Practice of Midwifery, or that charming volume, Watson's Practice of Physic, where the words and thoughts ripple along with mellifluous rhythm, and then labor through some of the ponderous tomes of to-day, my point of view would be quickly understood.

None of the various articles of manufacture and commerce are placed before the consumer without the expenditure of every effort to make them attractive. The current literature of the day is replete with all literary accessories that go to make it interesting. The popular magazines are seeking with avidity for scientific articles, that possess both worth and nicety of diction. It would seem, therefore that the medical writers might note with profit the trend of the times, and would find to their gratification that the small additional labor required to smooth and beautify their worthy offerings would produce a bountiful fruitage of grateful readers. N.

---

### PRIVILEGED COMMUNICATIONS.

It is not generally known among the profession that the confidences given to the doctor by the patient are subject to inquiry and investigation in the courts of Georgia. Physicians are not in court frequently enough to bring the matter to their attention and when they are, the questions asked are not always of a character to invade the confidential relations existing between them and their patients. But it remains true that a doctor may be questioned as to his knowledge of the most intimate facts of his patients' lives and be called upon under threat of fine and imprisonment to divulge what has been given to him in the most sacred confidence. Nothing is secure from the prying questions of counsel animated by a desire to win his case by any legal means and more or less schooled to the cruelty and mean practice of harrassing and even degrading an opposing witness in order to weaken or set aside the testimony he may be giving.

Legal technicalities have brought many a court into low esteem among the people who cannot understand the reason why facts should not be plainly stated and only such facts adduced as bear sensibly upon the matter in hand. Many lawyers keep themselves within these bounds in relation to the medical profession and never forget that the doctor is the recipient of confidences more intimate and complete than those given to the prelate and lawyer. These men respect the faith in which the confidences were given and deem it outside the bounds of decency to question them. But there are men who so far forget themselves as to invade or attempt to invade recesses they should never approach.

The Justice in charge has no option in Georgia; he must require an answer from the doctor if the question can be shown in the remotest way to be relevant, and the doctor must betray his patient or go to jail.

The communications between a client and a lawyer are privileged. That is, a lawyer can say that he will not answer any question because the information he has was acquired in his professional capacity. He has only to say this and the matter is dropped. The same is true of the minister or priest. People would be shocked if either of these men divulged what they received in professional confidence and anyone pressing them to do so would be scorned.

The Medical Profession should be relieved by law from this incubus resting upon its members. Every Doctor should feel that he when summoned, can go on the witness stand and tell the truth as a good citizen and at the same time be protected from questions that make him, if answered, a traitor to relations of sacred significance and importance. No criminals will receive protection or immunity thereby; neither will justice be perverted or strained, for every man qualified to judge as to life and death learns the importance of his position in the community and comes inevitably to discharge the responsibilities resting upon him as a good citizen should.

Indeed, the lawyers and priests, already privileged as to their testimony, protect criminals by the very nature of their calling far more than any physician would.

We urge that every physician in Georgia take such steps as seem to him wise toward having a law passed making the communications given to him by his patients, privileged from the Courts. This is a condition in many other States and it should exist here.

